# Coronavirus Disease 2019 Regulatory Response in United States-Assisted Living Communities: Lessons Learned

**DOI:** 10.3389/fpubh.2021.661042

**Published:** 2021-05-19

**Authors:** Sarah Dys, Jaclyn Winfree, Paula Carder, Sheryl Zimmerman, Kali S. Thomas

**Affiliations:** ^1^School of Public Health, Oregon Health & Science University-Portland State University, Portland, OR, United States; ^2^Institute on Aging, College of Urban and Public Affairs, Portland State University, Portland, OR, United States; ^3^Cecil G. Sheps Center for Health Services Research, University of North Carolina at Chapel Hill, Chapel Hill, NC, United States; ^4^School of Public Health, Brown University, Providence, RI, United States; ^5^Providence VA Medical Center, Providence, RI, United States

**Keywords:** socioecologic model, regulatory response, multilevel, thematic analysis, public policy, long-term care

## Abstract

Coronavirus disease 2019 (COVID-19) has disproportionately affected residents, their families, staff, and operators of congregate care settings. Assisted living (AL) is a type of long-term care setting for older adults who need supportive care but not ongoing nursing care and emphasizes a social model of care provision. Because AL is a type of long-term care, it has at times been referenced along with nursing homes in discussions related to COVID-19 but not recognized for its different care practices that pose unique challenges related to COVID-19; in that manner, it has largely been left out of the COVID-19 discourse, although ~812,000 older adults live in AL. To identify COVID-19 issues specific to AL, stakeholders with expertise in AL operations, policy, practice, and research (*n* = 42) were recruited to participate in remote interviews between July and September 2020. Using a thematic analysis, we derived the following overarching themes: (1) Policymakers are disconnected from and lack an understanding of the AL context; (2) AL administrators were left to coordinate, communicate, and implement constantly changing guidelines with little support; (3) AL organizations faced limited knowledge of and disparate access to funding and resources; (4) state-level regulatory requirements conflicted with COVID-19 guidelines resulting in uncertainty about which rules to follow; and (5) AL operators struggled to balance public health priorities with promoting their residents' quality of life and well-being. To develop evidence-informed policy and avoid unintended consequences, AL operators, direct care workers, residents, and clinicians practicing in these settings should have opportunities to provide feedback throughout the policy development process, both state and national.

## Introduction

The coronavirus disease 2019 (COVID-19) pandemic has disproportionately affected older adult residents of long-term care settings, who comprise 1% of the United States (U.S.) population but nearly 40% of COVID-19-related deaths (1). Broadly, long-term care settings include nursing homes (NHs), care homes, assisted living and residential care (hereafter assisted living/residential care referred to as AL). As of September 2020, of the estimated 82,105 COVID-19-related deaths in long-term care settings, 30% were linked to AL residents (2). Another recent study reported that one in five AL residents who tested positive for COVID-19 died, compared with a rate of 1 in 40 in the general U.S. population (3). Approximately 28,900 AL settings provide care to ~812,000 residents across the country, and nearly one-quarter of residents have an average of four chronic conditions (4). In addition, an estimated 42 to 72% of AL residents live with moderate/severe dementia or cognitive impairment (5). Given that dementia is a risk factor for COVID-19 mortality (6, 7) and AL residents live in a long-term care environment, COVID-19 prevention and response in AL is an important topic. Our primary goal was to understand issues unique to AL related to pandemic response, lessons learned, and aspects of AL that make it easier or more difficult to implement infection control policies and practices. Second, we sought to inform future policy development and regulatory response to emergencies, disasters, and other large-scale events that may impact AL. In this study, we learned from the lived experience of various stakeholders how the COVID-19 policy response materialized in and affected AL settings across the U.S.

It is important to understand issues specific to COVID-19 in AL because these settings differ from NHs in terms of regulatory oversight, staffing levels, direct care staff training, inspections and enforcement, and emergency preparedness regulations (8, 9). Generally, AL is a supportive living environment with a philosophy of social engagement, lack of national regulations, high prevalence of dementia, limited medical and nursing provider presence and direct care staff training, and a reliance on family members to supplement care (10). AL residents vary regarding their care needs; some residents may be fairly independent, whereas others receive assistance with multiple activities of daily living and chronic health conditions (11). AL settings can provide different levels of care based on residents' care needs in addition to housing and services to promote social engagement (12, 13). Also, whereas NHs are required to have licensed nurses available to provide skilled nursing care 24 h daily (9), most states restrict the provision of skilled nursing services in AL but permit external health providers to provide nursing/medical care onsite (14). One key difference is that AL is regulated at the state level, and therefore what is considered AL, the population AL serves, and requirements for care that ALs provide vary within and across states (15). The wide variation between and within states in AL licensure types (14, 15), populations, care, staffing, and resources greatly affect these communities' ability to respond to an ongoing, novel, global pandemic (16).

During the early months of the pandemic, many AL communities had to adapt their internal working models in accordance with ongoing regulatory updates from federal, state, and local governments and agencies, although the guidelines were designed for NH (16–19). For example, the Centers for Disease Prevention and Control (CDC) and Centers for Medicare and Medicaid Services (CMS) issued COVID-19 guidelines pertaining to NHs and hospitals. Although the CDC added recommendations for AL communities to seek guidance from state and local officials, they continued to reference CMS' NH guidance as a resource for local officials (20). Some COVID-19 related federal policies and recommendations may not be feasible in practice for AL settings because, as Dobbs and colleagues described, AL settings face unique challenges in implementing federal guidance compared with NHs (21), largely due to their use of external healthcare and service providers to provide resident care (14), lower staffing levels and licensed staff availability (9, 22, 23), variable infection control requirements (24), and (in)ability to isolate and cohort COVID-19 positive residents (25).

AL must also respond to various levels of regulatory oversight related to COVID-19 from state agencies, county and local health departments, and policy changes within their own organizations. The federal government allocated funding to support individuals, families, and businesses adversely affected by the pandemic *via* the “Coronavirus Aid, Relief, and Economic Security (CARES) Act” (26); however, individual states were charged with disbursing CARES act funds. Governors approached pandemic response differently in their states through executive orders (17). Taking these state executive orders into consideration, counties, health departments, and municipalities were also developing COVID-19-related requirements related to visitation, testing, and mask-wearing within AL settings.

Given the government structure within the U.S., and the multiple levels of policy responses impacting ALs, residents, staff, and families, it is necessary to understand better how these regulatory actions are related in the context of a *system*. Federal/national, state/local, AL organizations, and communities represent different levels of policy or regulatory actions. Entities can collaborate or silo across system levels to facilitate or inhibit the implementation of public health policy and practice interventions (27, 28). In public health promotion and policy, socioecological frameworks describe how individuals are situated within their physical and social environments, which are further nested within and interact across larger societal structures (i.e., institutions and policies) (29–32). Regarding long-term care, multilevel factors including regulatory requirements, financial resources, mechanisms of healthcare delivery (e.g., onsite *vs*. offsite), staff availability and roles, and the balance of formal and informal caregiving all affect older adult residents (33–35).

We apply multilevel, socioecological framing to examine COVID-19 regulatory and policy response in U.S. AL settings, ultimately to discern lessons learned for the future. Specific to the AL context, we were informed by Kemp and colleagues' (35–37) “Convoys of Care” model, which conceptualizes how residents' care networks span individual, interpersonal, community, and environmental levels. Individuals, organizations, and institutions are situated within social and political structures, meaning that context shapes AL residents' “convoys of care,” extending from AL residents, residents' families, care staff, and AL settings and influenced by local and state agencies and the federal government (35–37).

**Figure 1:**
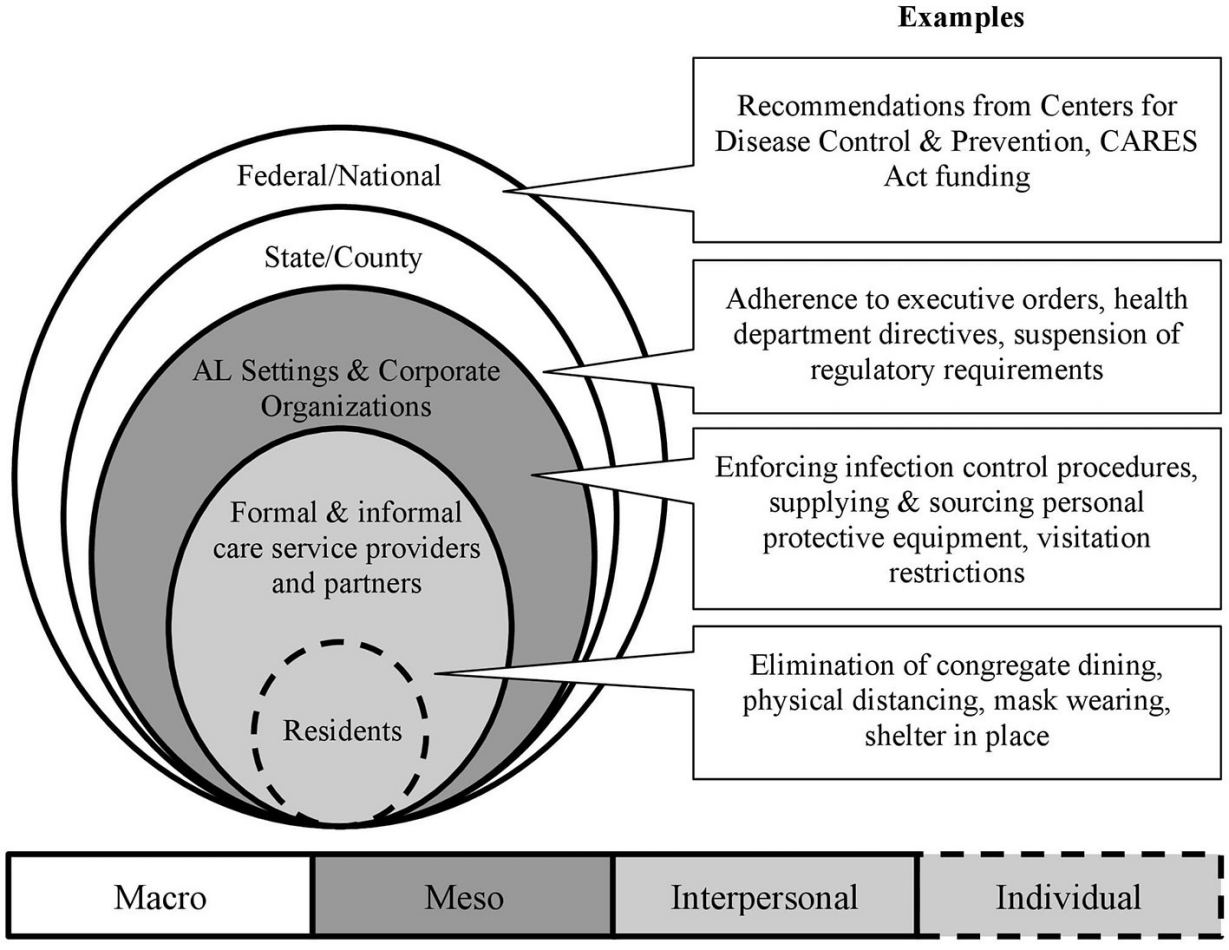
Levels of COVID-19 policy response related to Al.

Figure 1 presents the different levels of policy and regulatory response to the COVID-19 pandemic, as they relate to AL within a socioecological model. The first two concentric, shaded circles represent the microlevel or AL residents and their informal and formal care networks who may be most impacted by AL specific, county, state, and federal policies related to COVID-19. Residents and their formal and informal care partners, including AL care staff and administrators, external service providers (e.g., hospice, physical therapy), healthcare providers, and residents' families provide care within the AL setting. Furthermore, the policies enacted on and within AL settings may promote or inhibit the interrelationships and dynamics between these individuals. The outer circles represent the multilevel layering of meso-level (e.g., AL organizations, community) and macro-level (e.g., federal, state/county) policies that impact residents, their families, AL staff, and external care providers.

It is highly instructive to contextualize the AL residents and staff, AL settings, local and state agencies, and federal/national organizations as situated within a multilevel system because these levels are interrelated; just as a physician must be aware of what medications a patient is taking to avoid adverse reactions, so too must regulatory bodies be mindful of the effect of regulations on the system of care. Identifying the different levels and mechanisms of regulatory decision-making related to the COVID-19 pandemic response in AL can provide insights and lessons learned for a more efficient, less burdensome response for state regulators, AL operators, staff, residents, and residents' families that may inform responses during a future event with such large-scale effects.

## Methods

The research approach was exploratory and qualitative, as the topic—response to an international health crisis—continues to evolve. As is common with this approach (38), we sought diverse perspectives about AL setting-level experiences and state and federal actions. Thus, we recruited stakeholders with expertise related to AL regulations, operations, dementia care, and geriatric clinical practice. We first conducted an internet search for individuals working at or in collaboration with senior housing professional organizations, state agencies that license AL settings, and those who conduct research and clinically practice in AL settings as initial participants for this study (*n* = 10). Based on these initial interviews, professional contacts of the co-authors, and through a combination of purposive and snowball sampling, we ultimately emailed 100 stakeholders to participate in a 30–45-min virtual interview, and 43 agreed to do so. We conducted 41 interviews with 42 participants over telephone or Zoom between July and October 2020. One interview included two participants (denoted with the same interview ID and lowercase “a” and “b” to differentiate). One respondent had to withdraw from participating due to a scheduling conflict. Those who chose not to participate either did not have the time/capacity or did not respond to our requests. We continued purposeful recruitment of individuals to participate in interviews until we reached data saturation, determined by the authors through a reflexive process throughout data collection [see *Analysis*; (39–41)].

Our semi-structured interview guide was based on a question bank we developed that included various questions that interviewers could use depending on each participants' AL experience, especially as it pertained to COVID-19 prevention and response policies and practices. The interviewers asked clarifying questions and elicited examples based on both participants' initial responses and prepared prompts (please see Supplementary Table 1 for the full interview guide). For example, if an interviewee worked for an AL company, we asked questions about their company policies and staffing, but we did not ask these questions of long-term care researchers. Generally, the questions addressed COVID-19 responses, care coordination, regulatory changes, admissions/discharges, care transitions, telehealth, and lessons learned. Similar to other studies that use semi-structured interviews, the goal was to permit participants the opportunity to describe and reflect upon their experiences in their own words (42). This study received approval from the Portland State University Institutional Review Board (proposal #: 184614).

### Analysis

The first author (doctoral candidate, gerontology research assistant) and second author (gerontology research associate), both of whom have experience interviewing AL staff and stakeholders, conducted all interviews; one person led the interview, whereas the other transcribed participants' answers in real-time. The authors debriefed each other at the end of each interview and wrote key summaries that served as initial analytic memos. The first, second, and third authors (associate professor of public health and established expert in AL policy) met weekly to discuss interpretations of emerging patterns raised by participants *via* reflexive and annotated notetaking, which were used to guide analysis and determine when additional interviews did not yield novel codes or topics (39, 40, 43, 44). Using applied thematic analysis (41, 45, 46), we read the interview transcripts, developed codes, and categorized emergent themes with the underlying context of different levels of COVID-19 response (Figure 1). The purpose of this thematic analysis is to depict and describe the salient features captured within participants' narratives related to our focus on the multilevel nature of the regulatory response in these settings, rather than to quantify the frequency of topics raised (45–48). After reading each interview transcript, we applied codes based on the topics raised by participants and in response to our interview questions by hand and organized with Microsoft Excel. We coded text as relevant to policies, regulations, rules, restrictions, or guidelines connected to COVID-19 or infection control practices using codes that addressed resident experience, health service use, infection control, government agencies, state regulations, and AL policies and practices.

After initial coding, we reviewed coded sections of text to examine the levels of response to the pandemic indicated in the conceptual model: federal/national, state/county, and AL setting (Figure 1). We qualified text at the federal/national level if participants discussed national organizations and federal agencies or commented on the overall state of the long-term/healthcare systems. State/county-level text referenced specific states, state-level agencies, governors' executive orders, and local health or county authorities' directives. Participants' roles and affiliations were considered when qualifying text at the federal/national, state/county, or AL setting levels. Finally, we categorized text related to the AL setting if participants commented about policies, protocols, or practices within an AL building, community, or organization (i.e., two or more communities operated and/or managed by the same entity). Through this process of reading interview transcripts, coding, and code review, themes that described commonalities across multiple interviews were identified, and supporting quotes added to illustrate how regulations and policies at the AL setting (meso), state/county, and federal/national (macro) levels affected AL residents, residents' families, and staff according to the participants. The fourth and fifth authors are established health services researchers and full and associate professors (respectively) at their institutions; they provided reflexive feedback and contributed to honing theme specificity based on their expertise and scholarship in AL practices.

## Findings

### Participant Characteristics

The 42 participants' current affiliations included AL operators (*n* = 9), representatives of healthcare associations (*n* = 9), long-term care and health services researchers (*n* = 7), geriatric-trained clinicians (*n* = 7; registered nurses, nurse practitioners, and medical doctors), dementia care consultants/advocates (*n* = 6), and representatives of national advocacy organizations (*n* = 4). Notably, some of these individuals had prior and overlapping expertise, including a geriatrician who currently does dementia advocacy and a former AL operator and registered nurse who currently does policy work. Participants lived and worked in 16 states and the District of Columbia: California, Connecticut, Florida, Hawaii, Maryland, Minnesota, Mississippi, New Jersey, New York, Oklahoma, Oregon, Pennsylvania, Texas, Virginia, Washington, and Wisconsin. Ten participants served their organizations in a national capacity, so they provided perspectives beyond the context of the states where they physically live and work. Thus, the participants reflect a diverse range of stakeholders' perspectives on AL policy and practice.

### Themes

Stakeholders commented on overarching perceptions of AL as a care provider, difficulties responding to directives for various levels of government and agencies, and applying these directives within the AL context to meet the needs of residents, residents' families, and staff. We identified the following themes related to COVID-19 policy response in AL: (1) Policymakers are disconnected from and lack an understanding of the AL context; (2) AL administrators were left to coordinate, communicate, and implement constantly changing guidelines with little support; (3) AL organizations faced disparate access to funding and resources; (4) State-level regulatory requirements conflicted with COVID-19 guidelines resulting in uncertainty about which rules to follow; and (5) AL operators had to negotiate a balance of public health priorities with promoting their residents' quality of life and well-being. In the following paragraphs, we discuss each of these themes, including their subthemes and illustrative quotes from interview participants.

#### Theme 1: Policymakers Are Disconnected From and Lack an Understanding of the AL Context

Several stakeholders raised a general comment about AL as “disconnected from the rest of the system” within healthcare and long-term care across the nation, a reality exacerbated by COVID-19. As one dementia care expert and advocate (#20) shared, “[We] need political leaders to realize this segment of public health. [AL] is the public.” From stakeholders' perspectives, AL operators, senior housing professional organizations, and state agencies responsible for AL oversight were not given the opportunity to provide feedback or consultation when federal policymakers developed and issued initial guidelines and directives related to COVID-19. As a regional health services director for an AL organization (#7) described, “People do not understand what we do. We are kind of like the square peg in a round hole with these guidelines.”

Two salient subthemes emerged related to the perception and sense that policymakers were disconnected from the AL context. First, stakeholders expressed that guidance and discussion often focused on NH settings, and AL settings were assumed to be the same (Subtheme 1A). Relatedly, the second subtheme revealed that participants felt AL voices and perspectives were missing from policy development processes (Subtheme 1B).

*Subtheme 1A: A lack of understanding of AL as an independent entity from NH had regulatory consequences*. Assuming NH guidelines could be implemented within AL settings resulted in a missed opportunity for national and state policymakers to develop setting-specific guidelines based on regulatory and scope of practice differences between AL and NHs:

“It feels like AL has not gotten the attention it wants and needs and deserves. [...] CMS reg[ulation]s only apply to nursing homes, but we've heard from a lot of states that governors or state health departments have been plunking CMS reg[ulation]s on AL, so it's the same. It's just as restrictive in AL (National advocacy organization representative, #38).”

Stakeholders from New Jersey, New York, and Pennsylvania mentioned how AL settings were expected to meet the same infection control guidelines as NHs as issued by the Centers for Medicare and Medicaid services:

“They [ALs] did get cited if they failed to put things in place immediately. The mindset is “we are not NHs” and as true as that is you have a population that is similar to NHs so if AL had failure to [do] temperature checks, failure to report cases, failure to test, etc., they were cited. Some citations, though not as many, were related to donning PPE and cohorting measures between units. These citations are specific to recent directives that AL was not in compliance with CMS guidelines (Healthcare association representative, #8).”

In addition to the perceived confusion about the scope of practice within AL from federal agencies, AL operators, consumer advocates, and healthcare/trade association representatives remarked on how much time and effort they put forth explaining to local and state governments the nuance of AL policy and practice:

“The majority of phone calls with the governor's office, department of public health, and local health jurisdictions were to get them to better understand our industry. Oftentimes people lump AL with NHs, and they're not. [We] needed to share with those regulatory bodies how we staff, what the limitations are with the staff, buildings, etc. (Healthcare association representative, #26).”

*Subtheme 1B: Presence or absence of AL representation in policy development*. Like state regulations, COVID-19 response for AL settings varied between and within states. Some participants asserted that vague and non-prescriptive regulatory language allows AL settings to be creative in meeting the needs of their specific resident populations. One stakeholder from New Jersey (#3) commented, “None of our regulations say ‘must’ or ‘shall,’ they say ‘may’ and that is the word that gives us the flexibility to do what we need to do to take care of residents.” Combined with regulatory flexibility, some state agencies took a more collaborative approach in designing COVID-19 response policies to include the AL perspective and regulatory context, however, limited:

“[AL company] has a good reputation with state regulatory authority, so we submitted all our policies and procedures for their benefit, and we worked closely with New Jersey Hospital Association with a team of infection control specialists and epidemiologists to feed them the protocols we saw were working because they were involved with the state at the table. No [AL company] owner/operators had a seat at the table with the state to develop guidelines (Regional health services director for AL organization, #7).”

Those who discussed interdisciplinary collaboration as a feature of COVID-19 response also perceived better control of the pandemic:

“The model for good policy is collaboration. Everyone is in active conversations about all the concerns with PPE, testing, etc. The states that have put this kind of model in place are doing better than the states that have relied on historical rivalries between these [long-term care] settings and don't have strong relationships with the department of public health or strong understandings of emergency preparedness and that's a recipe for tragedy” (National professional organization representative, #11).”

#### Theme 2: AL Administrators Were Left to Coordinate, Communicate, and Implement Constantly Changing Guidelines With Little Support

Regulatory guidelines related to COVID-19 response were constantly updated, reissued, and came from different levels of governance (e.g., federal, state, county). Nearly every stakeholder mentioned how collecting, organizing, and disseminating frequent changes to AL operations to staff, residents, and residents' families created compounding challenges. In interviews, representatives of healthcare associations, policymakers, and AL operators remarked on the difficulty of collating the voluminous information coming from government agencies and regulatory bodies:

“There's a large quantity of non-synthesized information. There's a greater need for synthesis from the federal and state governments. There are instances where a federal change trumps a state provision, or a state piece has been undone (Healthcare association representative, #39a).”

Stakeholders who worked directly in AL operations, representatives of healthcare and trade associations, national advocates, and dementia care experts all remarked on the challenges and frustrations caused by confusion from entities attempting to provide guidance. When asked how various state agencies, local health authorities, and AL corporate organizations coordinated and communicated their infection control efforts, one AL operator (#40) shared that a key takeaway from caring for AL residents during a pandemic was “the importance of [AL] organizational leadership not counting [on] guidance from their state or federal overseers because that guidance was inconsistent or incorrect.” However, AL communities' abilities to assert their own agency are dependent on resource capacity. A participant who worked for an organization that operated multiple AL communities (#34) shared her experience with a community-level response,

“The home office made all the corporate policies. I feel sad for any really small facilities or people on their own. The admin[istrator] and staff have to concentrate on infection control and the normal business operation to keep things working. They don't have the time to keep up with the changes in guidance.”

Subtheme 2 focuses on a specific set of guidelines and the resulting confusion that nearly every participant touched upon: care transitions. Care transitions include the admission, discharge, and transfer of residents to and from various settings, including hospitals, post-acute rehabilitation settings, or back home with spouses or adult children. At the time of data collection, residents moving between the hospital and the AL setting posed distress for operators.

*Subtheme 2: Guidelines Related to Resident Admission, Discharge, and Transfer Caused Significant Concern to AL Operators*. Early in the pandemic, operators lacked clear guidance or protocols for transitioning residents to and from acute care settings that accounted for already existing congregate care requirements:

“Some of the biggest problems with other types of transitions are the return to community and what is required. It's getting better but we have a lot of folks who are stranded. The hospital wouldn't keep them, and the AL [community] wouldn't accept them back without a negative test. But [residents] have to go into hospitals to test people for COVID just so the AL [community] would take them back (AL clinical provider, #30).”

One healthcare association representative (#3) discussed how states mandated AL settings to take in new residents because of the volume of people who were COVID positive in hospitals,

“First, in New York, New Jersey, and Pennsylvania, at least those are the three states I know for sure, had directives from their governors who said skilled [nursing facilities] and AL must take residents back or new admissions regardless of COVID status. That was a disaster and many nurses spoke out about that. Even if AL [communities] were on the downswing with cases, they took such a surge due to these mandatory admissions.”

#### Theme 3: AL Organizations Faced Limited Knowledge of and Disparate Access to Funding and Resources

Access to additional resources to promote infection control practices in response to the pandemic was a key concern expressed by stakeholders from all levels. The resources most often described as inadequate were directly related to personal protective equipment (PPE), such as gloves, gowns, masks, and face shields. Many AL settings lacked access to PPE for their staff throughout the beginning of the pandemic as cases began to surge. AL settings provide care to a medically complex population of residents, yet there was a perceived hierarchy of access to supplies,

“PPE is a huge issue. It's not just the masks. You can't shower someone, provide close care without a gown. It's different than just delivering meals. You need to be gowned. It's been very difficult and expensive to get them. The NHs have been the first line to get support for PPE. AL is the next in line but you have a lot of people living with dementia in AL. It's important (Dementia care consultant and advocate, #36).”

Regarding financial support, a public policy consultant at a state healthcare association (#22) commented, “At the national level, we engage with AHCA/NCAL [American Health Care Association/National Center for Assisted Living] to support us with federal relief. AL is only regulated by states, and there are pros and cons to that. These [AL communities] are private entities, not federally regulated, and have not received any federal relief funds other Medicare providers have received.”

As testing became more widely available and guidelines were issued for AL residents and staff to receive regular tests, AL settings experienced additional financial challenges, which are novel to these settings. A healthcare association representative (# 8) noted, “Testing staff is a financial hardship because AL does not qualify for the federal funding or reimbursement that nursing facilities do. As state programs, national funding is not coming down to the AL settings.” Furthermore, testing capacity in AL presented a logistical challenge for settings to meet both recommended guidelines and mandates. For example,

“As CEO I had to create the response for following protocol. [The] state added some [protocols] along with CDC and CMS guidelines. For example, testing staff twice a week, residents once a week. Making sure we have all of the testing [supplies] we need. About 4,000 dollars a week to test employees. Testing to comply with the regulations. When someone [a resident] is quarantining you have to dress in full PPE, might be going in there to help with ADLs multiple times. It's 10 dollars each time, if you go in [a resident's room] 12 times a day, you can see how the expenses are really hard, you have to make sure you have funds coming in (AL operator, #39b).”

In addition to resource capacity, the lack of specific policies and protocols related to infection control within AL settings presented a practice and operational gap, described later in Subtheme 3.

*Subtheme 3: Infection Control Policies and Protocols in AL Need Improvement*. At the setting level, several stakeholders raised lack of access to and familiarity with specific infection control practices and using PPE as one of the largest barriers to successful COVID-19 response in AL:

“[AL settings] don't necessarily have the infection control procedures as much as we should. Communities were put in such a tough spot. [.] Need to make sure they [AL] have enough support and PPE. It's hard to do infection control if you have the same PPE for a week (National advocacy organization, #5).”

One long-term care researcher (#15) commented on the connection between the lack of resources and explicit infection control protocols,

“I guess that it's obvious that having infection control procedures in place ahead of time would be good. It's required, but it's not necessarily done within the facilities. Facilities may not be ready or have those procedures or supervision of staff to implement those procedures. I'm not being critical. I cannot imagine how these settings are dealing with this [pandemic]. I think many facilities did not have protective equipment they needed to have.”

State AL regulations lacked explicit specifications for setting-level infection control protocols and staff training related to infection control, which introduced an additional burden to pandemic response within AL. State-level requirements for infection control practices provided basic, general guidance:

“When I was looking into it I went down the rabbit hole of seeing who is regulated and who isn't. I found 27 states mention infection prevention and when it comes to hiring staff they just sign a statement regarding adhering to universal precautions. It's a big gap and even when flus come every year that can shut down a facility (AL clinical provider, #16).”

#### Theme 4: State-Level Regulatory Requirements Conflicted With COVID-19 Guidelines Resulting in Uncertainty About Which Rules to Follow

Regulatory challenges and contradictions inhibited AL settings' ability to comply with COVID-19 guidelines. In some circumstances, COVID-19 guidelines contradicted existing state AL regulations. As one AL operator and clinician (#4) described,

“State regulations were set up so that we cannot retain anyone with infectious disease. With the onset of COVID the state said “stop you have to keep them [residents with COVID/other infectious disease].” I am very involved with emergency preparedness; at county and state meetings I was always saying “look, there is a huge population of AL facilities that you do not have at the table and if there is a huge emergency, like a pandemic, they will not be able to handle it.” With COVID, my wildest nightmare came true.”

Another participant from Washington (#26) discussed the challenges that may result when AL representatives are not present in policy discussions and the difficulty in determining whose rules AL settings had to follow when guidelines did not match up with state licensing,

“Local health jurisdictions expected [AL] to follow CMS guidelines. We have companies that have facilities across the state having trouble writing cohorting policies. Yakima [county] may tell them this, when King [county] is saying that. [Communities] had to follow whatever the local health jurisdiction says. Licensing took a step back and the local authorities were telling everyone what to do without knowledge of AL.”

Additional directives related to COVID-19 and assumptions of AL scope of practice and capacity to carry out these directives impacted operations and staffing:

“For AL, the requirement was mandatory vital signs on every shift. AL [does] not have sufficient staffing to do that in New Jersey. Some AL [communities] were hiring staff to do nothing more than meet that requirement. The DPH [Department of Public Health] didn't understand that staffing ratios were not the same as in NHs (Healthcare association representative, #8).”

Furthermore, settings were put in a difficult position to maintain compliance with infection control guidelines, citing training, certification, or staffing level deficits. Stakeholders said, “[We] need to test on a regular basis, AL settings don't always have staff that's capable or a medical director who can write the orders” (healthcare association representative, #26) and “being scared of nursing shortages is not a reason to avoid testing people” (AL clinical provider, #29).

One physician (#33) who primarily served AL residents onsite shared how staffing levels impeded effective isolation, cohorting, and infection control practices within the AL setting:

“Staffing made everything worse because we didn't have any flexibility. We couldn't have just 2–3 staff for COVID-positive residents. If [a resident] got sick, [we] put everyone on super isolation, which was about the most we could do.”

#### Theme 5: AL Operators Had to Negotiate a Balance of Public Health Priorities With Promoting Their Residents' Quality of Life and Well-Being

AL operators discussed the negative effects of restrictions that prioritized mitigating the spread of coronavirus without considering long-term effects of social isolation for a vulnerable population, introducing consequences for residents:

“Residents would say “It's not COVID that's going to kill me, I'm more afraid of dying of these restrictions than COVID.” Some of the restrictions, [I] don't know if we're fully taking a public health perspective. Stopping COVID is important and we also need to look at the impact of isolation. State and county officials, [I] don't think they are at that point. They still have a risk mitigation, infection control perspective. As operators, who interact with residents and families every day, we see the impacts everyday more than investigators and epidemiologists. Regulations would benefit from a more whole person perspective during COVID (AL operator, #19).”

With many states and AL settings restricting visitation, mitigating the consequences of social isolation and finding creative ways to keep residents engaged presented additional adversity, especially for people living with dementia. An operator of a Mississippi continuing care retirement community (#32) shared,

“Isolation is different in memory care, can't get them to wear masks, hard with a small place and progression of disease. [We are] more liberal with them around their neighborhood. Window visits are difficult since it's such a high touch population. One resident didn't recognize her daughter. We could see how they lost that part of the connection with her daughter not being able to see her.”

From a policy and practice perspective, government entities and AL settings struggled to balance public health priorities and the physical and mental health consequences of restricting access to families and visitors for older adults living with cognitive impairment. One clinician trained in geriatric psychiatry commented,

“I've become really acutely aware of how bad the isolation has become and disruptive to routine. What I haven't seen yet nationally is any guidance on finding a happy medium. [.] Quarantine has long term mental health effects on people. We know this from other pandemics. I feel an urgency headed toward winter. Need some guidance around allowing family visits and restarting activities in a safe way. You cannot keep people locked in their rooms for months. It's cruel (Geriatric-trained psychiatrist, #23).”

A stakeholder from a national advocacy organization (#38) noted they “learned that you can kill people by trying to protect them. We have to put systems in place that don't pull people away from their relationships and connections and love that they need to survive.” Some stakeholders described creative adaptations to encourage engagement and community. One stakeholder from Hawaii (#22) described the need to balance infection control with the needs of residents as “collaborative but conservative.” Another clinician working in a continuing care retirement community (#1) mentioned various adaptations to maintain residents' engagement and socialization and families' connections to the goings-on within the community,

“There's enough space they can dine by themselves at a table and sit socially distanced. We've implemented Zoom for residents and families. Mostly for the families to check-in on us. Residents don't care about it. We have a marketing person that's an amazing singer; many residents say it's the highlight of their week. Highlight of my week too. Opportunities to see movies, things like that. Small group exercise, [we] maintained as much of that as we can.”

## Discussion

This study provides a window into the experiences of stakeholders associated with AL during the COVID-19 pandemic. Incongruent regulatory action at federal, state, and local levels combined with a lack of understanding of the AL context inhibited efficient pandemic response for settings, staff, residents, and residents' families. Participants discussed how this pandemic exposed infrastructure limitations and introduced new policy and practice paradoxes (49), resulting in a situation when two or more statements seem to be contradictory. At their inception, AL communities were designed to provide a social model for older residents who need support with activities of daily living and other services, as an alternative to the medicalized model associated with NHs and hospitals (12). Stakeholders described how the pandemic exposed gaps in states' regulations that buttress social and emotional support to residents when medical responses, such as PPE use, isolation, and quarantine, were necessary.

Participants highlighted how policymakers at all levels did not understand the heterogeneity of the AL context, which resulted in unfortunate consequences related to infection control compliance, accessing PPE, and meeting testing and cohorting requirements for staff and residents. Before the pandemic, the AL regulations of 32 states required settings to have infection control policies and staff training, although these requirements varied in specificity (24). Only two states explicitly mentioned pandemics in their AL regulations. Although AL communities can serve residents with significant care needs, the infrastructure of regulatory requirements and scope of practice might have influenced resource and operational capacity to respond to COVID-19-specific guidelines compared with licensed health settings.

Regulation and licensing of AL vary between and within states (15), as does the specificity of regulatory requirements (14). As noted by one stakeholder, state AL regulations might be vague, using the word “may” rather than “must,” which they interpreted as allowing AL providers great flexibility. From a policy perspective, states may intentionally create flexible policies by using vague language, although lack of regulatory specificity might also reflect inattention or oversight of a policy topic (50, 51). Some might see vague, non-prescriptive regulations, made more evident during the COVID-19 pandemic, as a reason for federal oversight and increased regulatory requirements in AL (52). However, developing and implementing federal practices and policies for AL communities is complex, political, and not universally endorsed. Among other reasons, proponents have argued that federal regulation would limit the diversity of AL that is fundamental to its model (53). Medicaid reimbursement in community-based care is one example that highlights the complicated nature of federal oversight of AL. State Medicaid agencies have eligibility guidelines that define who may receive Medicaid-funded services in AL, and state licensing agencies maintain oversight (54). Although CMS is a federal agency, they do not directly enforce or oversee rule compliance with AL communities that receive Medicaid dollars (55).

Stakeholders' descriptions of resident care transitions to and from hospital settings at times of surging cases presented a salient example of both a policy paradox and failure of interdisciplinary and multilevel interaction. Care transitions from ALs to hospital settings and other healthcare entities can create confusion and complications for administrators and residents, regardless of an active pandemic. In addition to practical and logistic ambiguity, many state regulations do not offer specific directives for these types of care transitions (14). However, some states explicitly restrict admission to prospective residents who have been exposed to infectious diseases. For example, Maryland's AL program regulations state: “An AL program may not provide services to individuals who at the time of initial admission, as established by the initial assessment, would require: Treatment for a disease or condition which requires more than contact isolation” (56). Throughout the pandemic, AL communities received mixed messages from regulators about the safety and capacity of hospitals and emergency departments (57). Participants described confusion as to whether or not to immediately discharge residents who tested positive for COVID-19 to hospitals. Transitioning residents back to their AL was further complicated by admission criteria (e.g., inability to admit residents with skilled nursing needs) and testing protocols. AL administrative participants expressed the desire to provide end-of-life care and testing to residents within AL settings, yet felt bound by regulatory complexity and contradictions.

All stakeholders involved directly or indirectly with COVID-19 response might benefit from understanding the nuanced context of AL settings within each state. Particularly, policymakers at all levels should be aware of including AL perspectives in AL policy development processes and the impact of intentional collaboration across levels of governance while balancing public health goals with quality of life and well-being for all residents. These contexts have implications for the scope of services provided to residents under the purview of AL and, therefore, their ability to respond to blanket guidelines extended to licensed health settings, including NHs. Federal requirements for AL communities to procure PPE neglected to understand the ways in which AL funding varies from NHs—namely that AL communities have different, if any, access to federal funding or financial reserves for that kind of necessity. Although AL is largely a private-pay industry, none have sufficient reserves for the financial burdens exacted by COVID-19. Investigating both success and failures of response to COVID-19 provides a learning opportunity for political, regulatory, and long-term care systems to develop mechanisms that increase collaboration and communication across sectors (28).

## Limitations

The findings in this study represent the views of the stakeholders who chose to participate (42% of those solicited) and are not representative of all states' responses to the pandemic. This study is limited by stakeholders recounting their experiences with AL policy responses, retrospectively and concurrently, in that the pandemic has not ended and policy responses remain dynamic and ongoing. It is possible that between the time of this writing and when stakeholders shared their experiences, federal, state, and/or local regulatory response to COVID-19 related to AL changed in significant ways. For example, in September 2020 (the end of the data collection period), the U.S. Department of Health and Human Services announced private pay AL settings qualified for funds appropriated by the CARES act, expanding access to federal support (58). The field may benefit from a more focused, longitudinal analysis examining policy approaches during the beginning of the pandemic and the extent to which federal and state governments addressed policy contradictions for AL over time. Additionally, the racial and ethnic disparities of COVID-19 morbidity and mortality seen in the community are mirrored in long-term care populations (59, 60). There is a need for more inclusion and centering of the experiences of AL residents, staff, and operators, including older adults coded as racial or ethnic minorities or who are members of the lesbian, gay, bisexual, and transgender community who have experienced systemic exclusion from these spaces. Lastly, although we recruited multilevel stakeholders for this study, our participants did not include direct care staff nor residents currently living in AL or their family members. It is possible that frontline care staff, residents, and residents' families might have different perspectives than those raised by the stakeholders in this study. Inclusion of resident, family, and direct care staff perspectives would have provided an additional layer to this contextual analysis of the individual and interpersonal impact and strengthened our study overall.

## Conclusion and Implications

The COVID-19 pandemic underscored the importance of understanding meaningful differences among long-term care settings; the need to centralize and collaborate to communicate changing recommendations and guidelines; that access to resources and funding affects adherence to guidelines, regulatory representation, and contradictions; and the need to balance public health response with residents' overall quality of life. Oversight and licensing at the state, county, and local levels introduce complexity making “one-size fits all” policy solutions currently infeasible, and lack of endorsement from national organizations make it unlikely.

Despite the immense challenges presented by the pandemic, the range of national, state, and local policymakers across the U.S. have an opportunity to engage in knowledge sharing and learn from the experiences of AL stakeholders across the country and within states when designing rules and regulations that have an impact on AL. To make evidence-informed policy and avoid unintended consequences, AL operators, direct care workers, residents, and clinicians working with AL populations should have opportunities to provide feedback at the policy-making table, both state and national. These perspectives can inform the design of policies and regulatory guidance that acknowledge the experience and expertise of those who live and work in AL during and beyond the immediate COVID-19 pandemic.

## Data Availability Statement

Data supporting the conclusions of this article with direct and indirect identifiers redacted will be made available upon request.

## Ethics Statement

The studies involving human participants were reviewed and approved by Portland State University Institutional Review Board (proposal # 184614). The patients/participants provided their written informed consent to participate in this study.

## Author Contributions

All authors contributed to developing the research question, conceptualization of the study, and development of the interview prompts and writing of the manuscript. SD and JW organized data collection (i.e., recruitment, interviews, and transcription), led analysis, and contributed to all written sections of the manuscript. PC mentored SD and JW as junior scholars and oversaw data collection, contributed to analysis and write up of methods, results, and discussion. SZ contributed to the framing and methods written sections, and theme development. PC and SZ served as a co-investigators on this research study funded by the National Institute on Aging. KT contributed to the introduction, theme development, discussion and was the principal investigator of this research study funded by the National Institute on Aging.

## Conflict of Interest

The authors declare that the research was conducted in the absence of any commercial or financial relationships that could be construed as a potential conflict of interest.
