# Supplementary material for: Coronavirus Disease 2019 Regulatory Response in United States-Assisted Living Communities: Lessons Learned
Source: Front Public Health. 2021 May 19;9:661042. doi: 10.3389/fpubh.2021.661042 (PMC8170034; doi:10.3389/fpubh.2021.661042)
Supplement: Supplementary file 1 [file Table_1.DOCX]

Supplement Table 1. COVID-19 question prompts used to facilitate semi-structured interviews.

| Topic | Question |
| --- | --- |
| Introduction | Can you begin by describing your current role or roles as they relate to assisted living settings and/or older adults in congregate care? |
| General | What was your role specific to COVID-19 response in your state/organization? [e.g., technical assistance, coordinate prevention activities, interpret policies for AL staff, resident care] |
|  | Can you walk us through some of the first activities that your state/organization took specific to COVID response in assisted living settings? |
|  | How are things going now, several months since the pandemic first started? |
|  | Did you learn anything from other states’ responses that you found helpful? |
|  | Many issues have been raised by this pandemic, including the availability and capacity for testing and contact tracing, the risk of social isolation among residents, visitation policies, maintaining adequate staffing levels, and getting reliable information about the disease. How has your agency/organization addressed these issues in terms of assisted living? What other issues have come up that were unexpected, or unique to assisted living? |
|  | For this study, we are especially interested in the care of people living with dementia and other forms of cognitive impairment. What are the challenges of COVID-19 specific to assisted living residents living with these conditions? |
|  | Is there anything you can add about how the COVID-19 pandemic has impacted diverse residents, including those who are Medicaid beneficiaries, and people from under-represented groups, including racial and ethnic minorities and the LGBTQ community? |
| Care coordination, providers, and staff | Are you aware of current corporate policies to support assisted living administrators during COVID-19? If yes, can you describe? |
|  | How are direct care staff trained to provide care for residents during COVID-19? |
|  | How often should staff be tested or retested for COVID-19? How about residents? |
|  | Are assisted living settings having trouble providing personal protective equipment for their staff? |
|  | How are assisted living settings coordinating end-of-life care and planning or residents who have been diagnosed with COVID-19? |
|  | What are the different ways of cohorting residents who are suspected to be exposed or have been diagnosed with COVID-19 to keep other residents and staff safe? |
| Regulations | How would you describe the state of infection control and communicable disease policies in assisted living before the pandemic? |
|  | To what extent are ALs being cited for regulatory deficiencies in the area of infection control or staffing specific to COVID? |
|  | Since the pandemic, how have the various agencies and organizations involved in assisted living, including licensing agencies, public health agencies, and AL owners or corporate offices, coordinated their efforts? Can you give an example? |
|  | How has the state communicated with assisted living operators during the pandemic? |
| Admissions, discharges, and care transitions | How have admission/discharge policies changed as a result of the COVID-19 pandemic? |
|  | How are assisted living settings handling care transitions of residents to and from the hospital? |
| Telehealth | How have assisted living settings used technologies to manage COVID-19? This might include adding or increasing telehealth capabilities as well as strategies for communicating with residents’ families, technologies to promote social interaction among residents, or other ways that technology has been used since the pandemic started. |
| Conclusion | What are some of the lessons learned about caring for assisted living residents during a pandemic? This might include lessons about residents, their families, staffing, and preventing or responding to outbreaks. |
|  | To conclude, is there anything about assisted living that makes it easier or more difficult to implement infection control policies in comparison to other congregate or licensed health care settings? |
|  | Is there anything else we should know or anything you would like to mention that we did not address? |
